# A multi-head self-attention deep learning approach for detection and recommendation of neuromagnetic high frequency oscillations in epilepsy

**DOI:** 10.3389/fninf.2022.771965

**Published:** 2022-09-09

**Authors:** Xiangyu Zhao, Xueping Peng, Ke Niu, Hailong Li, Lili He, Feng Yang, Ting Wu, Duo Chen, Qiusi Zhang, Menglin Ouyang, Jiayang Guo, Yijie Pan

**Affiliations:** ^1^Information Technology Research Center, Beijing Academy of Agriculture and Forestry Sciences, Beijing, China; ^2^National Engineering Research Center for Information Technology in Agriculture, Beijing, China; ^3^Australian Artificial Intelligence Institute, Faculty of Engineering and Information Technology, University of Technology Sydney, Ultimo, NSW, Australia; ^4^Computer School, Beijing Information Science and Technology University, Beijing, China; ^5^Department of Radiology, Imaging Research Center, Cincinnati Children's Hospital Medical Center, Cincinnati, OH, United States; ^6^Department of Radiology, Jiangsu Province Hospital of Chinese Medicine, Affiliated Hospital of Nanjing University of Chinese Medicine, Nanjing, China; ^7^Department of Magnetoencephalography, Nanjing Brain Hospital, Affiliated to Nanjing Medical University, Nanjing, China; ^8^School of Artificial Intelligence and Information Technology, Nanjing University of Chinese Medicine, Nanjing, China; ^9^The Affiliated Hospital of Medical School, Ningbo University, Ningbo, China; ^10^National Institute for Data Science in Health and Medicine, Xiamen University, Xiamen, China; ^11^Department of Hematology, School of Medicine, Xiamen University, Xiamen, China; ^12^Department of Computer Science and Technology, Tsinghua University, Beijing, China; ^13^Ningbo Institute of Information Technology Application, Chinese Academy of Sciences, Ningbo, China

**Keywords:** high frequency oscillations (HFOs), magnetoencephalography, MEG, deep learning, multi-head self-attention, HFOs detection, HFOs recommendation

## Abstract

Magnetoencephalography is a noninvasive neuromagnetic technology to record epileptic activities for the pre-operative localization of epileptogenic zones, which has received increasing attention in the diagnosis and surgery of epilepsy. As reported by recent studies, pathological high frequency oscillations (HFOs), when utilized as a biomarker to localize the epileptogenic zones, result in a significant reduction in seizure frequency, even seizure elimination in around 80% of cases. Thus, objective, rapid, and automatic detection and recommendation of HFOs are highly desirable for clinicians to alleviate the burden of reviewing a large amount of MEG data from a given patient. Despite the advantage, the performance of existing HFOs rarely satisfies the clinical requirement. Consequently, no HFOs have been successfully applied to real clinical applications so far. In this work, we propose a multi-head self-attention-based detector for recommendation, termed MSADR, to detect and recommend HFO signals. Taking advantage of the state-of-the-art multi-head self-attention mechanism in deep learning, the proposed MSADR achieves a more superior accuracy of 88.6% than peer machine learning models in both detection and recommendation tasks. In addition, the robustness of MSADR is also extensively assessed with various ablation tests, results of which further demonstrate the effectiveness and generalizability of the proposed approach.

## 1. Introduction

About 30% of pediatric patients with epilepsy are medically intractable and require respective neurosurgery to gain seizure freedom (Durnford et al., 2011; Yamakawa et al., 2020). Recording epileptic activities are crucial to the pre-operative localization of epileptogenic zones and the optimization of the diagnosis of epilepsy. The success of epilepsy surgery depends on the pre-operative localization of epileptogenic zones (Guo et al., 2018). Although intracranial electroencephalography (iEEG) is commonly treated as the gold standard for the localization of epileptogenic zones, it may bring a risk of infection and bleeding during implantation (Hu et al., 2015). Thus, a noninvasive detection method for epileptogenic zones is preferred to epilepsy surgery. Magnetoencephalography (MEG) is a noninvasive technology for the detection of epileptic activities. MEG has a higher spatial resolution to localize epileptic activities for epilepsy surgery than other noninvasive approaches, such as electroencephalography (EEG) (Nakasato et al., 1994).

Localizing epileptogenic zones play a central role in epilepsy surgery. However, to date, there are no robust biomarkers that are able to accurately capture the location of epileptogenic zones (Tamilia et al., 2017). A variety of diagnostic indicators are introduced in the current clinical practice to estimate the epileptogenic zones. Despite the progress, existing methods, which heavily rely on epileptic spikes (typically ≤ 70 Hz), fail to reduce seizure frequency in approximately 50% of the cases, which greatly limits their applications in epilepsy surgery (Stigsdotter-Broman et al., 2014; Olan Çocuklarda and Öncesi, 2015; Oldham et al., 2015; Reinholdson et al., 2015; Verdinelli et al., 2015). High frequency oscillations (HFOs) (typically 80-500 Hz) can be used to localize the epileptogenic zones as biomarkers. Recent studies (Xiang et al., 2010; Ontario, 2012; Modur, 2014; Van Klink et al., 2014; Van't Klooster et al., 2015; Leung et al., 2018; Nevalainen et al., 2020) show that applying pathological HFOs to localize the epileptogenic zones leads to a significant reduction in seizure frequency, even seizure elimination in about 80% of cases. Thus, pathological HFOs have been associated with epileptogenic zones (Xiang et al., 2009; Miao et al., 2014). There is increasing evidence to show that HFOs are putative biomarkers to identify epileptic regions of the brain, which may improve the surgical diagnosis and surgical outcomes of patients with epilepsy.

Recent reports (Papadelis et al., 2009, 2016; Van Klink et al., 2016; Von Ellenrieder et al., 2016; Hedrich et al., 2017; Fan et al., 2021; Guo et al., 2022) have shown that MEG can detect epileptic spikes and HFOs effectively. In the presurgical diagnosis process, it is critical to accurately detect the HFOs in MEG signals for improving the post-surgical outcomes of patients with epilepsy. Visual reviews of HFOs in MEG signals by human experts play an important role in current clinical practices. However, visual identification of HFOs is usually subjective, time-consuming, and error prone due to the large volume of MEG signal data (Zelmann et al., 2012; Roehri et al., 2017; Fujiwara et al., 2020). Consequently, a number of automatic approaches (Gardner et al., 2007; Zelmann et al., 2010; Jacobs et al., 2012; Burnos et al., 2014) has been proposed to enable HFO detection so as to assist human experts for the visual review of iEEG and MEG signals. During the detection tasks, a universally two-step framework is applied by most of these methods: (1) The whole recording data is divided into a large number of signal segments. (2) The HFO detectors extract certain signal features for decision making. The handcrafted features that are manually designed based on observation or statistical analysis play as the solution for the feature of HFO signals. For example, Van Klink et al. (2017) proposed an automatic HFO detection and visualization approach in MEG. Similarly, in another work (Burnos et al., 2014), handcrafted features (e.g., high frequency peak and low frequency peak) were proposed to automatically distinguish HFOs in EEG signals. In these works, a cutoff for handcrafted features is often required to recognize an HFO signal segment. It is clear that these approaches based on handcrafted features require to be adjusted or re-optimized when the detectors are applied to similar neuroimaging data from different populations. This circumstance hinders the generalizability of HFOs in unseen conditions. Recently, machine learning provides a possible opportunity for improving the performance of HFO detections and reducing human interference. Traditional machine learning algorithm (Elahian et al., 2017), such as logistic regression, has been used for the identification of the epileptogenic zones. More recently, a deep learning approach SMO detector (Guo et al., 2018) was proposed. Such deep learning based HFO detector requires minimal human interference by using a golden standard dataset to train the detector.

Objective and automatic detection of HFOs in MEG signals with advanced deep learning algorithms may serve as a promising clinical decision support system to assist human experts for the visual review of MEG signals (Guo et al., 2018; Kong et al., 2019). In addition to the correct detection of HFOs in the MEG signals, recommending the possible results to clinicians is also crucial for an accurate and timely clinical evaluation. The recommended HFO list may not only serve as evidence for the particular patients but also serve as clinical diagnosis cases for future data retrieval purpose (e.g., teaching and research). In this study, our overall goal is set to develop a deep learning model to detect HFO signals with high confidence among a large amount of MEG data and recommend these findings to clinicians as a clinical decision support system. To that effect, we propose a multi-head self-attention deep neural network as the HFO detector. Compared to the existing algorithms (e.g., SMO detector Guo et al., 2018), we introduce the popular multi-head self-attention mechanism in this paper to enable an HFO detector to jointly pay attention to important information from various representation subspaces at multiple positions. Instead of computing the attention once, this multi-head self-attention strategy is able to compute the importance of each feature multiple times in parallel. Our hypothesis is that an HFO detector with multi-head self-attention mechanism is able to outperform the existing detector based on deep neural networks. Our newly developed HFO detector enables clinicians to objectively and automatically observe and localize HFOs for the preparation of epilepsy surgery without human designed signal features. According to the output probability values of our proposed detector, the MEG signals can be sorted in descending order. In order to accurately and timely understand the patient's condition, we can recommend *N* signals with the highest HFO signal probability value (e.g., top-10) to the clinician and assist in developing a treatment plan. This process is also known as a top-*N* recommendation task (Zhao et al., 2013, 2014; Wang et al., 2017; Zhang et al., 2017).

The following sections of this paper are organized as follows: First, we describe the patients and their associated MEG data in this work and the detailed MSADR framework in the Section 2. Second, experiment setups, such as model evaluation, peer machine learning models, and developmental environment are described. Third, we present the performance of detection and recommendation of MSADR for HFO signals. Then, ablation studies are also conducted to test our MSADR approach. Fourth, the discoveries and limitation of this work are discussed. Finally, we conclude the paper by summarizing the contributions and future directions.

## 2. Materials and methods

### 2.1. MEG data

#### 2.1.1. Data acquisition

In this retrospective study, we obtained interictal MEG data from 20 clinical patients with epilepsy consisting of 10 females and 10 males (age: 6–60 years, mean age: 32 years), who were affected by focal seizures arising from one part of the brain. The Institutional Review Board was approved, and written informed consents were obtained from all subjects.

Full details of MEG data acquisition can be found in our prior study (Guo et al., 2018). Briefly, MEG recordings were performed using a 306-channel, whole-head MEG system (VectorView, Elekta Neuromag, Helsinki, Finland) in a magnetically shielded room. Sleep deprivation and reduction of anti-epileptic drugs were used to increase the chance for capture HFOs during MEG recordings, as one part of the pre-surgical evaluation. An approximate 1 h of MEG data was recorded for all patients. The sampling rate of MEG data was 2,400 Hz. The noise floor in our MEG systems was calculated with MEG data acquired without a subject (empty room). The noise floor was used to identify MEG system noise. The noise level was about 3–5 fT/Hz. The empty room measurements were also used to compute the noise covariance matrix for localizing epileptic activities (i.e., HFOs). A three-dimensional coordinate frame relative to the subject's head was derived from these positions. The system allowed head localization to an accuracy of 1 mm. The changes in head location before and after acquisition were required to be less than 5 mm for the study to be accepted. To identify the system and environmental noise, we routinely recorded one background MEG dataset without patients just before the experiment.

#### 2.1.2. Segment

**Figure 1:**
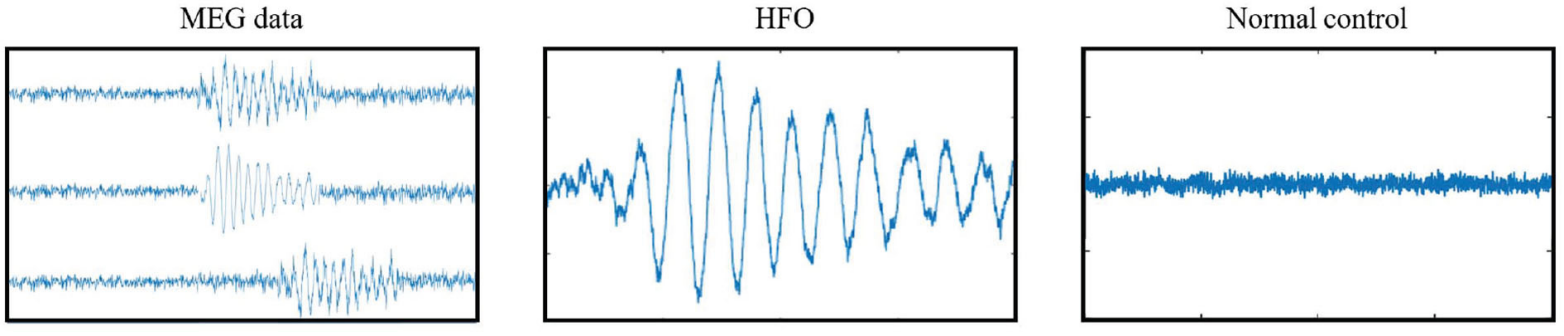
Examples of gold standard signals.

A public available software MEG Processor (Xiang et al., 2009) was used to correct and label the MEG data. In the current work, the MEG data were segmented into about 11,016,000 signal segments^1^ with 2 s window size without overlap. Each signal segment was a MEG signal intensity vector with 4,800 data points in the time domain. These segments were first filtered automatically, which segments with a goodness-of-fit value of <85% or confidence volume of >3mm^3^ were dropped. Second, two band-pass filter, an 80–250 Hz one for ripples and a 250–500 Hz one for fast ripples, were introduced to filter high frequency MEG data into candidate segment set while the low frequency ones were automatically dropped. Both physiologic and pathologic high frequency neuromagnetic signals were included in the candidate segment set. The physiologic HFOs were manually rejected, and then the pathologic ones were selected by comparing MEG ripples and iEEG recordings at source levels (Wu et al., 2014) by two human experts. A total of 660 HFO signal segments selected by human experts, together with 660 normal control (NC) signal segments randomly selected from the rest segments, were compiled into our gold standard dataset. Figure 1 shows examples of MEG data, HFO, and NC segments.

### 2.2. Method

#### 2.2.1. Overview framework for HFO detection and recommendation

**Figure 2:**
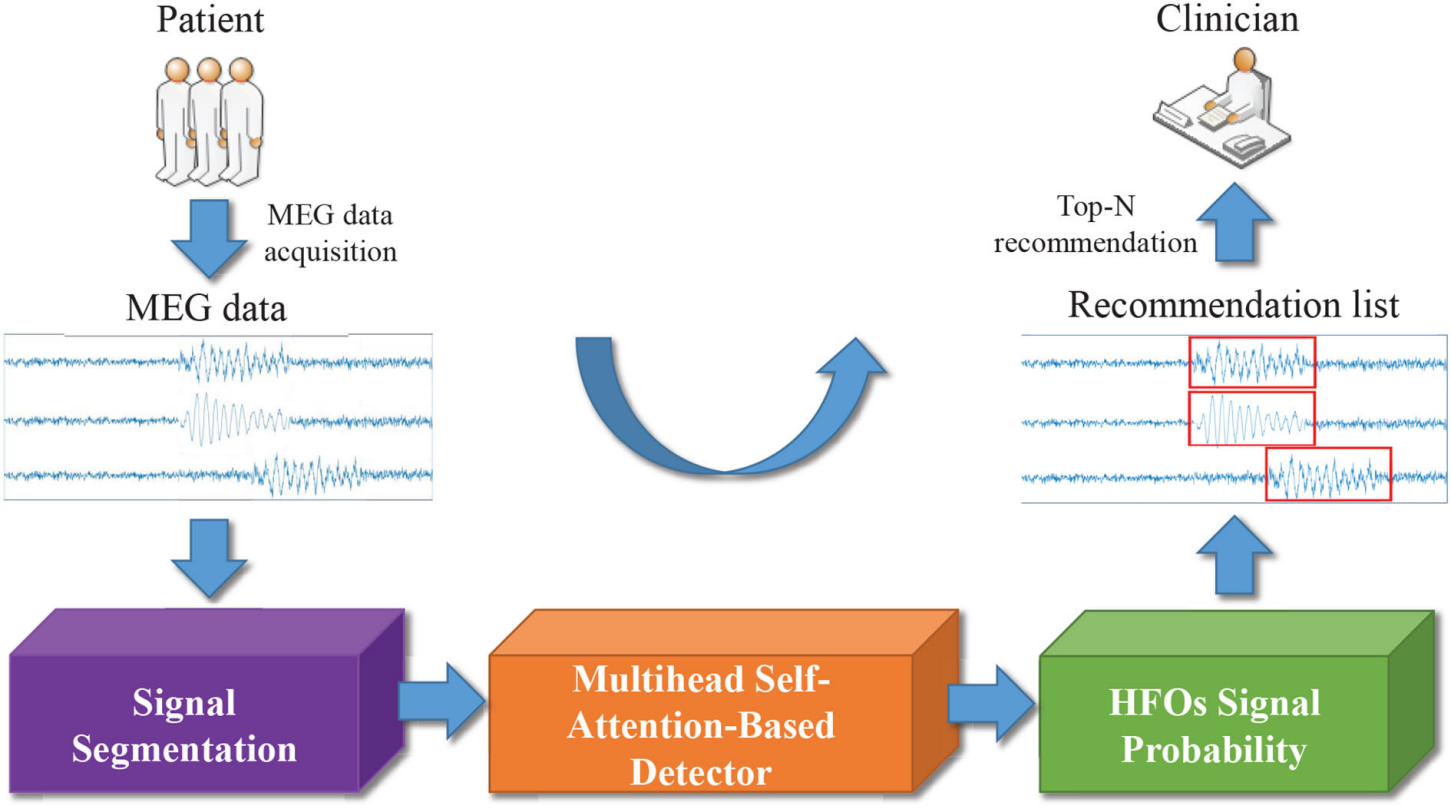
Overview of multi-head self-attention based detector for recommendation of neuromagnetic high frequency oscillations in epilepsy.

In Figure 2, we display the overview of our multi-head self-attention based detector for recommendation (MSADR), which consists of MEG data acquisition, signal segmentation (purple box), multi-head self-attention based detector (orange box), HFO signal probability (green box), and detection and recommendation list.

**Figure 3:**
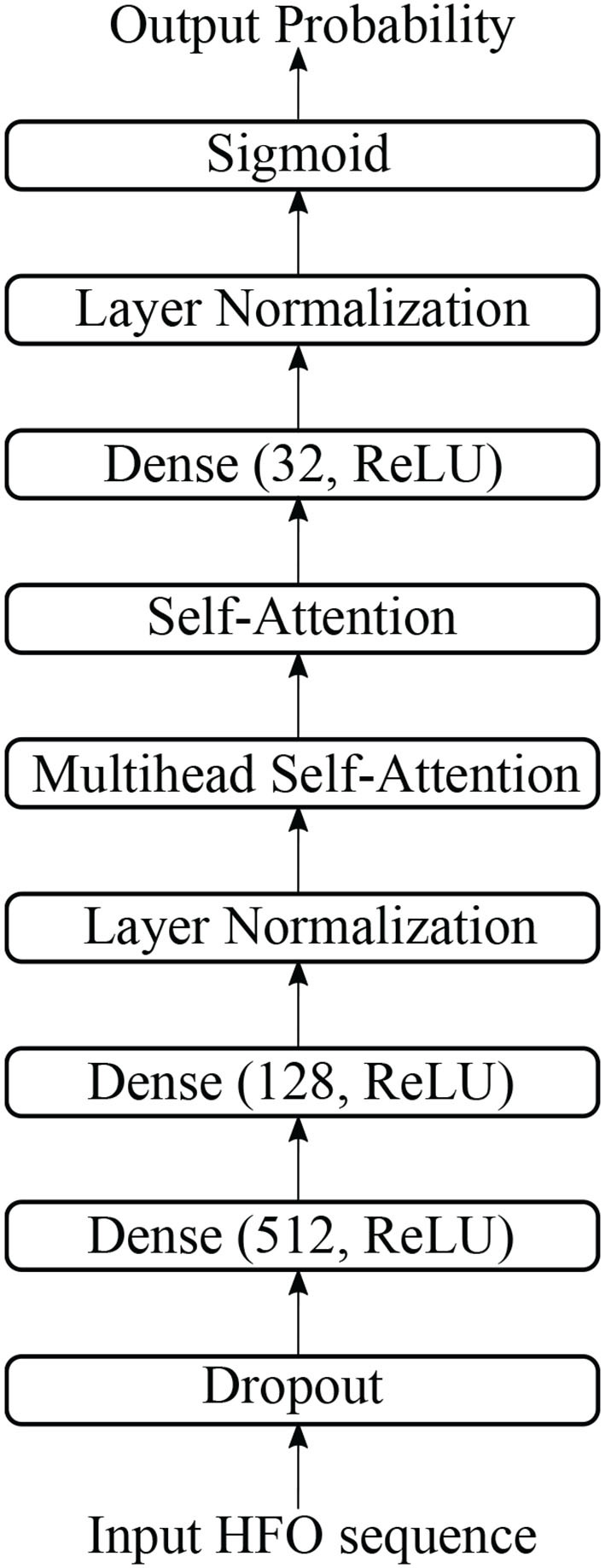
The structure of multi-head self-attention-based detector in this study.

The structure of multi-head self-attention based detector (MSAD) is given in Figure 3. It consists of layers of dense, normalization, multi-head self-attention, self-attention. The various components are described in the following sections.

#### 2.2.2. Dropout, dense, and normalization

**Figure 4:**
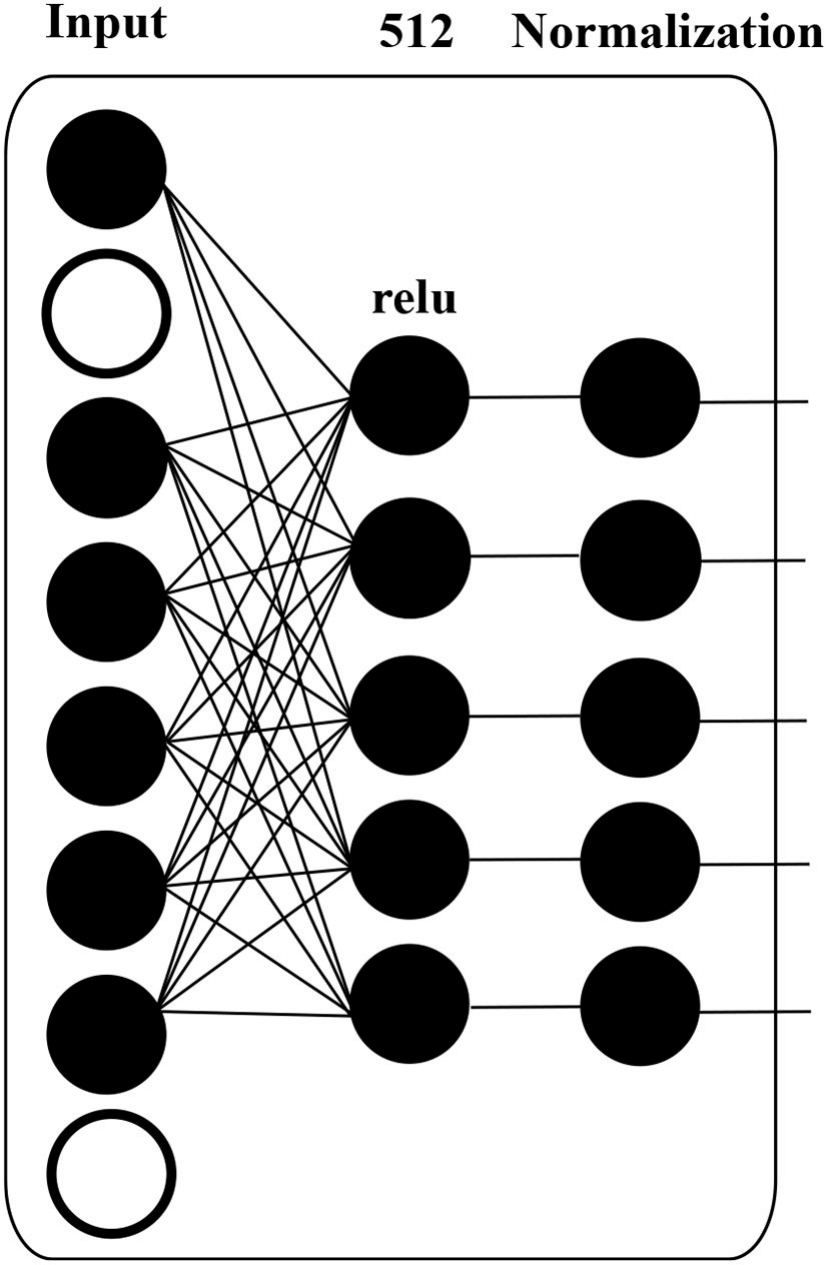
Details of MLP block in DANN structure.

There is one dropout layer, three dense layers, and two normalization layers in our proposed model. Figure 4 shows the computation details of these layers.

A dropout layer, which prevents over-fitting during model training, is applied to input data, i.e., HFO sequence. The white circle in Figure 4 indicates dropped units according to dropout probability. The dropout layer is followed by a dense layer, whose hidden units are 512, and the activation function is “relu”, to reduce the dimension of the previous layer. The normalization layer (Ioffe and Szegedy, 2015) is used to accelerate deep network training by reducing internal covariate shift.

#### 2.2.3. Self-attention

The attention is proposed to compute an alignment score between elements from two sources (Shen et al., 2018). In particular, given a sequence of HFOs, *x* = [*x*_1_, *x*_2_, ..., *x*_*n*_] and a representation of a query *q* ∈ ℝ^*d*^, the attention computes the alignment score between *q* and each element *x*_*i*_ using a compatibility function *f*(*x*_*i*_, *q*). A softmax function then transforms the alignment scores ${\left[f\left(x_{i},q\right)\right]}_{i=1}^{n}$ to a probability distribution *p*(*z*|*x, q*), where *z* represents the importance degree to *q*. That is, a large *p*(*z* = *i*|*x, q*) means that *x*_*i*_ contributes important information to *q*. This attention process can be formalized as follows:


(1) $$\begin{array}{l} \alpha ={\left[f\left(x_{i},q\right)\right]}_{i=1}^{n}, \end{array}$$

(2) $$\begin{array}{l} p\left(z=i|\text{x},q\right)=softmax\left(\alpha \right). \end{array}$$

The output *Attention* is the weighted element according to its importance, i.e.,


(3) $$\begin{array}{l} Attention\left(q,\text{x}\right)=p\left(z=i|\text{x},q\right)\text{x}. \end{array}$$

Additive attention (Bahdanau et al., 2015; Shang et al., 2015) is a commonly-used attention mechanism where the compatibility function *f*(·) is parameterized by a MLP, *i.e*.:


(4) $$\begin{array}{l} f\left(x_{i},q\right)=w^{T}\sigma \left(W^{\left(1\right)}x_{i}+W^{\left(2\right)}q\right), \end{array}$$

where *W*^(1)^ ∈ ℝ^*d*×*d*^, *W*^(2)^ ∈ ℝ^*d*×*d*^, *w* ∈ ℝ^*d*^ are learnable parameters, *d* is the number of columns of *x*_*i*_, and σ(·) is an activation function. Compared with multiplicative attention (Rush et al., 2015; Sukhbaatar et al., 2015) using cosine similarity or inner product as the compatibility function for *f*(*x*_*i*_, *q*), *i.e*.:


(5) $$\begin{array}{l} f\left(x_{i},q\right)=\langle W^{\left(1\right)}x_{i},W^{\left(2\right)}q\;\rangle , \end{array}$$

Though additive attention is expensive in time cost and memory consumption, it achieves better empirical performance for downstream tasks.

Self-attention (Liu et al., 2016; Lin et al., 2017; Peng et al., 2021) explores the importance of each feature to the entire HFOs given a specific task. In particular, *q* is removed from the common compatibility function which is formally written as the following equation:


(6) $$\begin{array}{l} f\left(x_{i}\right)=w^{T}\sigma \left(W^{\left(1\right)}x_{i}\right), \end{array}$$

(7) $$\begin{array}{l} \alpha ={\left[f\left(x_{i}\right)\right]}_{i=1}^{n}, \end{array}$$

(8) $$\begin{array}{l} p\left(z=i|\text{x}\right)=softmax\left(\alpha \right). \end{array}$$

The output *Attention* is the weighted element according to its importance, i.e.,


(9) $$\begin{array}{l} Attention\left(\text{x}\right)=p\left(z=i|\text{x}\right)\text{x}. \end{array}$$

#### 2.2.4. Multi-head self-attention

Multi-head self-attention allows the model to jointly attend to information from different representation subspaces at different positions. We use the multi-head version with *k* heads, as introduced in Vaswani et al. (2017),


(10) $$\begin{array}{l} MultiHead\left(\text{x}\right)=Concat\left(head_{1},\ldots ,head_{k}\right)W^{\left(O\right)}, \end{array}$$

(11) $$\begin{array}{l} \text{where}\;head_{i}=Attention\left(\text{x}W^{\left(x\right)}\right), \end{array}$$

where projections using learned parameter matrices *W*^(*x*)^ ∈ ℝ^*d* × *d*/*k*^, and *W*^(*O*)^ ∈ ℝ^*d* × *d*^.

#### 2.2.5. Loss function

A standard cross-entropy loss is used as the training objective of MSADR, defined as


(12) $$\begin{array}{l} L=-y\log\left(p\right)-\left(1-y\right)\log\left(1-p\right), \end{array}$$

where *y* is the target label (0 or 1) and *p* is the predicted probability between 0 and 1 given an HFO sequence.

## 3. Experiment setup

We evaluate the proposed model on two tasks including the classification of whole patients and recommendation for each individual patient.

### 3.1. Model evaluation

We conduct a comprehensive evaluation in this study by employing the proposed MSADR on the HFO dataset to classify HFO data and recommend detected HFO sequences to medical experts. We employ the evaluation strategy of leave-one-out cross-validation in our experiments. In the 10-fold leave-one-out cross-validation, the HFO dataset is separated to two parts. One consists of 90% of the whole as training data while the rest part is regarded as test data.

For the detection task, we put all segments from 20 patients together to separate the training data and test data. We first calculate true positive (TP), false positive (FP), true negative (TN), and false negative (FN) by comparing the predicted labels and gold-standard labels. Then, we calculate accuracy, recall, precision, and F-score by


$$\begin{array}{l} Accuracy=\frac{TP+TN}{TP+TN+FP+FN},\\ Recall=\frac{TP}{TP+FN},\\ Precision=\frac{TP}{TP+FP},\\ F-score=2\times \frac{Precision\times Recall}{Precision+Recall}. \end{array}$$

For the recommendation task, the dataset is separated according to patients, in each split, segments from 18 patients are selected into the training set while segments from the rest 2 patients as test data. We use top-*N* precision (P@*N*) (Choi et al., 2016) to evaluate the ability of the algorithm to recommend detected HFOs for individual patients in the test set, defined as follows:


$$\text{P}@N=\frac{\text{TP}@N}{N},$$

where “TP@*N*” in the formula stands for TP HFOs in top-*N* recommendation task. Top-*N* precision mimics the behavior of doctors conducting differential diagnoses, where doctors list most probable diagnoses and treat patients accordingly to identify the patient status. Therefore, a machine with a high top-*N* precision translates to a doctor with an effective diagnostic skill. This makes top-*N* precision an attractive performance metric for our problem (Choi et al., 2016).

### 3.2. Peer machine learning models

To compare our proposed model MSADR with existing machine learning models, we also implemented random forest (RF) (Breiman, 2001; Nissen et al., 2018), support vector machine (SVM) models (Ak et al., 1999; Zhang et al., 2020), and SMO detector (Guo et al., 2018).

- **Random Forest (RF)**: RF is a classic ensemble learning methods by learning multiple decision trees and employing averaging to improve classification performance and control over-fitting. The number of trees in the forest was optimized from empirical values [20, 40, 60, 80, and 100]. We set maximal depth of the tree as 10.
- **Support Vector Machine (SVM)**: A SVM model is developed to perform classification by using vectorized FC features. We apply a linear kernel and search for the margin penalty with empirical values [0.2, 0.4, 0.6, 0.8, and 1.0].
- **SMO detector (Guo et al.**, **2018****)**: In terms of the existing deep learning model, we compared our model with SMO detector, a DNN model developed previously for HFO detection. Briefly, we implemented the SMO detector model as a 7-layer DNN, with input number of HFO sequence in the input layer, followed by dropout, dense (512, ReLU), dense (128, ReLU), normalization, dense (32, ReLU), normalization, and the sigmoid layer to generate one output unit. A cross entropy loss function is applied to supervise the network learning adopted. The learning rate is set as 0.0001. A total of 10 epochs are applied to ensure the convergence of the model.

### 3.3. Developmental environment

The proposed DANN and peer machine learning models are all implemented in Python 3.7 environment. To build the deep learning related models, we apply TensorFlow (2.0.0-rc1) backend. For the traditional models, we adopt the models from Sklearn 0.20.2.

All the experiments are conducted on a workstation with 10 cores of Intel Core i9 CPU and 64GB RAM. Due to the high computation cost of deep learning algorithm, we use one GPU (Nvidia TITAN Xp, 12GB RAM) to accelerate the training speed of the models.

## 4. Results

We evaluate the performance of detection and recommendation for each set of experiments. There are two sets of experiments to be conducted, which consist of overall performance compared with baseline models and effectiveness of varying head number of multi-head self-attention.

### 4.1. Overall performance comparison

#### 4.1.1. Detection

We first compare the HFO detection performance of the proposed MSADR model and multiple peer machine learning models, including RF, SVM, and SMO. The results are derived on a leave-one-out cross-validation experiment by using the entire dataset. As shown in Table 1, our proposed MSADR takes the lead place (the bold value) in all metrics of HFO detection accuracy (0.886), recall (0.840), and F-score (0.859) among compared machine learning models, while the RF model returns the lowest detection performance on recall and F-score, and SVM on accuracy. Our model outperforms the SVM model by 0.126 on accuracy, the RF model by 0.263 on recall, and 0.142 on F-score.

**Table 1:** Detection comparison of random forest (RF), support vector machine (SVM), SMO, and multi-head self-attention-based detector for recommendation (MSADR) trained using leave-one-out cross-validation on the entire dataset.

**Method**	**Accuracy**	**Recall**	**Precision**	**F-Score**
RF	0.779	0.577	**0.951**	0.717
SVM	0.760	0.743	0.764	0.753
SMO detector (Guo et al., 2018)	0.845	0.732	**0.951**	0.826
MSADR	**0.886**	**0.840**	0.881	**0.859**

The bold values represent the lead place of the corresponding metric.

#### 4.1.2. Recommendation

We then compare the HFO recommendation performance of the proposed MSADR model and baseline models including RF, SVM, and SMO. The experiment setting is almost the same as the detection task, except for an additional recommendation module to generate a ranking list. As shown in Table 2, our proposed MSADR obtains the best HFO recommendation performance on P@1 (0.967), P@3 (0.858), and P@5 (0.879) among the compared machine learning models, whereas the RF model returns the lowest recommendation performance on P@3, P@5, and SVM on P@1. Our model increases the performance of SVM by 0.1 on P@1, the RF model by 0.162 and 0.215 on P@3 and P@5, respectively.

**Table 2:** Recommendation comparison of RF, SVM, SMO, and MSADR trained using leave-one-out cross-validation on the entire dataset.

**Method**	**P@1**	**P@3**	**P@5**
RF	0.893	0.696	0.664
SVM	0.867	0.800	0.760
SMO detector (Guo et al., 2018)	0.893	0.811	0.793
MSADR	**0.967**	**0.858**	**0.879**

The bold values represent the lead place of the corresponding metric.

#### 4.1.3. Computational costs

The computational costs of the proposed MSADR model and baseline models are provided in Table 3. It is noticed that our proposed MSADR takes a longer time for model training and inference than baseline models. The main reason is the complexity of the deep learning models (MSADR and SMO) and the huge number of the parameters. The MSADR has 411,325 parameters to be trained, which obtains a stronger learning capacity to get the best model performance. MSADR uses a learned model to conduct the detection and recommendation task, which theoretically take more time.

**Table 3:** Computational cost comparison of RF, SVM, SMO, and MSADR.

**Method**	**Training time (ms)**	**Inference time (ms)**	**No. of parameters**
RF	284	6	-
SVM	83	5	2,400
SMO detector (Guo et al., 2018)	2,506	140	318,449
MSADR	12,317	525	411,325

The results in Tables 1, 2 also show a trend that deep learning models (MSADR and SMO) achieve improved performance compared to the traditional model, such as SVM and RF, demonstrating the superior capability of the deep learning model on complex data patterns, such as HFO. In addition, the inference time of MSADR is about 0.5 s (525 ms). This is an acceptable time cost while it can bring about 16.6% accuracy improvement on detection and 11.5% P@1 improvement on recommendation toward the fastest model (SVM). Another trend can be observed that the P@1 returns the best recommendation score a cross all models, which demonstrates that the machine learning model is a promising alternative approach to assist clinicians to make decisions.

### 4.2. Effectiveness of varying head number of multi-head self-attention

The effectiveness of our MSADR is further tested by varying head number (*k* = [2, 4, 8, 16]) of multi-head self-attention on two tasks of detection and recommendation. The results in this set of experiments are calculated based on leave-one-out cross-validation by using the entire dataset.

#### 4.2.1. Detection

**Figure 5:**
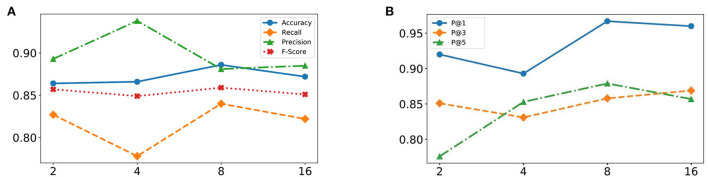
Effectiveness of varying head number of multi-head self-attention from 2 to 16. **(A)** Detection task. **(B)** Recommendation task.

The HFO detection performance of the proposed MSADR model has been evaluated by varying head number of multi-head self-attention. Figure 5A displays plots of the accuracy, recall, precision, and F-score of the proposed MSADR over different strategies of varying head number.

It is apparent that the proposed MSADR achieves the state-of-the-art in terms of accuracy, recall, and F-score when the head number is set as 8, while the performance on precision is the worst. Performance accuracy, recall, and F-score decrease as the head number increase due to the over-fitting of self-attention, whereas performance on precision slightly increases. Overall, the proposed MSADR has the best three indicators out of four when the head number of multi-head self-attention is set to 8.

#### 4.2.2. Recommendation

The effectiveness of the proposed MSADR model over HFO recommendation task has been compared in terms of P@*N* by varying head number of multihead self-attention. Figure 5B displays plots of the P@1, P@3, and P@5 of MSADR over different strategies of varying head number. As shown in the figure, our proposed MSADR achieves the highest HFO recommendation performance on P@1 and P@5, while the performance of the two indicators decreases as the head number increases due to the over-fitting of multi-head self-attention. Overall, the proposed MSADR has the best two indicators out of three when the head number of multi-head self-attention is set to 8.

### 4.3. Ablation study

A detailed ablation study is performed to examine the contributions of the model's components to the tasks of detection and recommendation. There are four configurations of replaceable components in this model. The two components are (1) multi-head self-attention layer and (2) the self-attention layer. The four configurations based on MSADR are

- **raw (DNN):** (1) and (2) are removed from MSADR, which becomes a pure DNN, one of our peer baseline models (SMO detector);
- **Attn_1:** (2) is removed and (1) is remained in MSADR;
- **Attn_2:** (1) is removed and (2) is remained in MSADR;
- **MSADR:** our proposed model.

All models are trained with 10 epochs and a batch size of 32. The head number of multi-head self-attention is empirically set to 8.

#### 4.3.1. Detection

From Table 4, we find that the MSADR model obtains the best performance on detection task compared to the ablated models, except for the performance of Attn_1 on precision. Moreover, we note that Attn_1 and Attn_2 outperform raw, which gives us the confidence to apply self-attention to learn the relationship between HFO signals. It is clear that the single self-attention model provides comparable information to the performance of the Attn_1 and Attn_2 model. In particular, MSADR outperforms the best ablated model for Accuracy by 1.1%, for Recall by 2.1%, and for F-score by 1.0%.

**Table 4:** Detection comparison of ablated models trained using leave-one-out cross-validation on the entire dataset.

**Method**	**Accuracy**	**Recall**	**Precision**	**F-Score**
raw	0.845	0.732	0.951	0.826
Attn_1	0.845	0.755	**0.975**	0.849
Attn_2	0.875	0.819	0.928	0.847
MSADR	**0.886**	**0.840**	0.881	**0.859**

The bold values represent the lead place of the corresponding metric.

#### 4.3.2. Recommendation

Table 5 shows the recommendation performance for the ablated models and our proposed model. As can be seen from the table, the proposed model achieves the best performance compared to the ablated models on the recommendation task. We observe that Attn_1 and Attn_2 outperform raw on P@1 and P@3, which again demonstrates that self-attention is a vital component to learn the relationship between HFO signals. MSADR outperforms the best ablated model by 4.7%, 1.1%, and 0.6% on P@1, P@3, and P@5, respectively.

**Table 5:** Recommendation comparison of ablated models trained using leave-one-out cross-validation on the entire dataset.

**Method**	**P@1**	**P@3**	**P@5**
raw	0.893	0.811	0.793
Attn_1	0.913	0.844	0.759
Attn_2	0.920	0.847	0.873
MSADR	**0.967**	**0.858**	**0.879**

The bold values represent the lead place of the corresponding metric.

## 5. Discussion

Since first discovered in the 1990s, HFOs have been considered a promising biomarker to locating the seizure onset zone and improving postsurgical outcomes in patients with epilepsy (Huang and White, 1989; Fan et al., 2021). Noninvasive brain recording technologies (i.e., scalp EEG and MEG) were a milestone in human HFO research and have provided the possibility to investigate this brain activity in a wider range (Papadelis et al., 2009, 2016; Van Klink et al., 2016; Von Ellenrieder et al., 2016; Hedrich et al., 2017). Due to excellent temporal resolution and acceptable spatial resolution, MEG is able to effectively record HFOs and localize epileptic activities for epilepsy surgery (Fan et al., 2021). After noninvasive recording, the detection of HFOs is the next crucial task for onset zone detection. Although visual identification is still considered to be the gold standard for HFO detection, it still faces the problem of highly time-consuming and subjective (Frauscher et al., 2017).

This study mainly focuses on the automatic detection and recommendation of HFOs from interictal MEG data. The MEG data of clinical epileptic patients were recorded with a multi-channel whole-head MEG system (Xiang et al., 2010; Guo et al., 2018), and then segmented into signal segments with 2 s window size without overlap. The labeled HFO segments by human experts and randomly selected NC segments from the complementary set of labeled HFO set was compiled into our gold standard dataset. With the gold standard data, we trained the proposed MSADR algorithm for the detection and recommendation model of HFOs. For a new patient, the trained model can detect HFOs from the segmented MEG data and recommend HFO signals to clinicians, alleviating the burden on reviewing the large amount of MEG data. The effectiveness of our proposed detection and recommendation approaches were demonstrated by the cross-validation experimental results. The proposed MSADR can improve the detection accuracy by at least 13.7% and the top-1 recommendation precision by 8.2% compared with the traditional machine learning methods (RF and SVM) while improving the detection accuracy by 4.8% and the top-1 recommendation precision by 8.2% compared with another deep learning method (SMO detector). The computational costs are important, especially in real world applications. Though MSADR is a time-consuming method, the acceptable inference time (0.5 s) can guarantee the user experience. In addition, sliding window with overlap can be used for segmenting, so as to improve the possibility of HFO locating in the center of segments in real world applications.

There are some limitations to this study. First, the experiment was built on a small dataset with 20 patients. A larger data set is required to further validate the effectiveness and efficiency of MSADR. Second, this work only focused on the detection and recommendation of HFOs from interictal MEG data. Performance of the MSADR approach on other neuromagnetic data (i.e., ictal MEG, iEEG, and EEG) remains unclear. We will test our method in future work. Third, the MEG segments from different patients or channels are treated equally and independently in this paper. However, there are complex timing and co-occurrence relationships among segments. Mining and utilizing these relationships may improve the effectiveness of HFO detection and recommendation. Finally, since the current approach requires signal segmentation of MEG data, it is only able to differentiate HFOs and NC segments with a pre-defined fixed signal length. It cannot directly detect HFOs in an automatic way on the raw MEG data (e.g., start and end positions).

Due to the high cost and lack of automatic detection technology with broad applicability, traditional MEG has limited availability (Guo et al., 2018; Kong et al., 2019). However, the technological innovations in MEG have been progressing. New MEG systems with optically pumped magnetometers do not require cooling with liquid helium and can be worn more conveniently (Boto et al., 2019). This may reduce the cost of MEG data recording and expand the scope of application. The research and application of automatic or semi-automatic HFO detection methods with broad applicability will make more efficient use of MEG data (Guo et al., 2018), and the integration into clinical review software can effectively enhance clinical value, including preoperative localization of epileptogenic regions, the assessment of disease severity, predicting seizures, monitoring treatment, evaluating treatment effects, and assessing epileptic susceptibility after brain injury (Fan et al., 2021).

## 6. Conclusion

In this study, we develop an MSADR detector for the detection and recommendation of HFO signals by using the multi-head self-attention mechanism. By comparing our model with traditional machine learning models (RF and SVM) and deep learning model (SMO detector), the proposed MSADR detector is proved to reach state-of-the-art performance in both detection and recommendation tasks. The robustness of our detector is also extensively assessed with multiple ablation tests. The MSADR is supposed to detect HFOs from a large amount of segmented MEG data and recommend HFO signals to help clinicians locate epileptogenic regions and assist in treatment. Our future directions may focus on extending our model to capture the timing and co-occurrence relationships among segments to further improve the effectiveness of HFO detection and recommendation.

## Data availability statement

The datasets presented in this article are not readily available because of a confidentiality agreement that prevents them from being disclosed to the public. Requests to access the datasets should be directed to JG, guojy@xmu.edu.cn.

## Ethics statement

The studies involving human participants were reviewed and approved by Nanjing Brain Hospital. The patients/participants provided their written informed consent to participate in this study.

## Author contributions

XZ, XP, KN, JG, and YP contributed to the conception and design of the study. XP and FY developed the DL algorithms. TW made a contribution to data collection. XZ, DC, QZ, MO, and JG performed the statistical analysis. XZ and KN wrote the initial draft of the manuscript. All authors contributed to manuscript revision, read, and approved the submitted version.

## Funding

This work was supported by the National Key Research and Development Program of China (Nos. 2020YFD1100605 and 2019YFD1101100), Beijing Information Science and Technology University Qin Xin Talents Cultivation Program (No. QXTCP C202112), Research Level Improvement Project (No. 2020KYNH214), Beijing Educational Science Planning Project of China (No. CHCA2020102), National Institutes of Health (Nos. R01-EB029944 and R01-EB030582), National Natural Science Foundation of China (Nos. 82172022 and 62006100), 2025 Key Technology Innovation Program of Ningbo City (No. 2019B10082) Fundamental Research Funds for the Chinese Central Universities (0070ZK1096 to JG), and Ningbo Public Welfare Technology Plan Project (No. 2019C50081).

## Conflict of interest

The authors declare that the research was conducted in the absence of any commercial or financial relationships that could be construed as a potential conflict of interest. The reviewer AM declared a shared affiliation with the author TW to the handling editor at the time of review.

## Publisher's note

All claims expressed in this article are solely those of the authors and do not necessarily represent those of their affiliated organizations, or those of the publisher, the editors and the reviewers. Any product that may be evaluated in this article, or claim that may be made by its manufacturer, is not guaranteed or endorsed by the publisher.

## Footnotes

^1^The number of segments is calculated as 20(*patients*) × 306(*channels*) × 60(*min*) × 60(*s*) ÷ 2(*s*/*window*).
